# A new species of *Argyromys* (Rodentia, Mammalia) from the Oligocene of the Valley of Lakes (Mongolia): Its importance for palaeobiogeographical homogeneity across Mongolia, China and Kazakhstan

**DOI:** 10.1371/journal.pone.0172733

**Published:** 2017-03-22

**Authors:** Paloma López-Guerrero, Olivier Maridet, Zhaoqun Zhang, Gudrun Daxner-Höck

**Affiliations:** 1 Geologisch und paläontologische Abteilung, Naturhistorisches Museum Wien, Vienna, Austria; 2 Jurassica Museum, Collection Management Center, Porrentruy, Switzerland; 3 Department of Geosciences, Earth Sciences, University of Fribourg, Fribourg, Switzerland; 4 Key Laboratory of Vertebrate Evolution and Human Origin of Chinese Academy of Sciences, Institute of Vertebrate Paleontology and Paleoanthropology, Chinese Academy of Sciences, Beijing, China; Institute of Botany, CHINA

## Abstract

We describe a new species of Rodentia (Mammalia), *Argyromys cicigei* sp. nov. from Toglorhoi (fossil bed TGW-A/2a) in Mongolia and Ulantatal (fossil beds UTL 1 and UTL 7) in China. Its tooth morphology differs from the type species *Argyromys aralensis* from Akespe in Kazakhstan by smaller size and simpler structures. *Argyromys* has been assigned in different families of Muroidea, such as Tachyoryctoididae and Spalacidae. However, the presence of common characters indicates a closer relationship of *Argyromys* with the genera of Cricetidae s.l. (subfamilies Eucricetodontinae; Cricetopinae; Cricetodontinae and Gobicricetodontinae among others) from Asia than with the earliest representatives of Spalacidae or the endemic Tachyoryctoididae. *Argyromys cicigei* sp. nov. possesses a simple anterocone and anteroconid in the upper and lower first molars, respectively, which is characteristic for Cricetidae s.l. It has a flat occlusal surface in worn specimens; weakly-developed posterolophs; an oblique protolophule and metaloph on the upper molars and it lacks a labial anterolophid on the m1. These traits are also typical of the Oligocene genera *Aralocricetodon* and *Plesiodipus*, included in the subfamilies Cricetodontinae and Gobicricetodontinae respectively. The cladistic analysis performed here supports this hypothesis. The clade formed by *Argyromys* species is grouped with other cricetid taxa (s.l). Spalacids, however, form a different clade, as do the tachyoryctoids. Previous authors state that the Aral Formation (Kazakhstan) should be dated to the Oligocene instead of the Miocene, based on the presence of several taxa. The finds of *Argyromys* in both regions supports the statement that they are closer in age than previously thought. The occurrence of *Argyromys* in Kazakhstan, Mongolia and China evidences the biogeographic unity of the Central Asian bioprovince during the Oligocene.

## Introduction

The superfamily Muroidea (Rodentia) is well-known for being the most diversified group of mammals [1–3]. The large number of known Cenozoic small mammal fossil sites provides a wide range of information about systematics and palaeobiology of the extinct representatives [4–13]. Within the Muroidea, those rodents with myomorphous zygoma and three cheek teeth are commonly recognized either as the family Cricetidae [14–19] or the family Muridae [1, 13]. We follow the authors that maintain a separate status for the Cricetidae and Muridae families [3]. Nevertheless, Cricetidae present several dental patterns and thus a number of subfamilies are recognized within it such as: Eucricetodontinae, Cricetodontinae, Cricetopinae and Gobicricetodoninae among others. We will use the term Cricetidae sensu lato (s.l.) to refer to the family Cricetidae in the sense of Musser and Carleton [2] and include the subfamilies recognized by Mein and Freudenthal [4]; Ünay [6]; Freudenthal [20]; McKenna and Bell [1] and De Bruijn et al. [13].

Among muroid rodents, the Cricetidae s.l. faunas are essential for biogeographic and phylogenetic studies [21–25]. The oldest occurrences of cricetid-grade muroids features correspond to genera recovered from the middle Eocene in Asia, mainly China [26] and Kazakhstan [27–32]. The richest cricetid records are from the Neogene [15, 33–36], whereas the transitional Oligocene remains are not yet fully documented. It is also worth noting that Mongolia, being geographically near China and Kazakhstan, is a key area regarding the origin of Cricetidae s.l. Recently, researchers have shown an increasing interest on the Oligocene cricetids s.l. of Asia [15, 37–41] providing a new understanding of the evolutionary process of Cricetidae s.l. in Eurasia.

Since 1995, extensive sampling has been carried out in the Taatsiin Gol and Taatsiin Tsagaan Nuurf areas, which are part of the Valley of Lakes region in Mongolia. More than forty localities spanning a time range from the early Oligocene to the late Miocene have been investigated. The age determination of the fossil-bearing sequences is based on the biostratigraphy of small mammals (biozones A–E) and on radiometric ages (^40^Ar/^39^Ar) of interlayered basalts [42–44]. Part of the rodent fauna from the Oligocene and Miocene of Mongolia has been previously studied [38, 42–47]. The present study is focused on a new species description from the Taatsiin Gol area in Mongolia [10, 16, 48]. It is found within the biozone C (late Oligocene) from the Taatsiin Gol area assigned to *Argyromys* [49]. Only one species—*Argyromys aralensis* [50]—had been described within this genus. The type locality is situated in the North Aral Region, Akespe locality, Aral Formation in Kazakhstan. The suprageneric classification of *Argyromys* has been controversial. There are multiple proposals made by different authors. The fossils reported here provide additional morphological information that could help to clarify its taxonomic position.

### Stratigraphy

**Fig 1:**
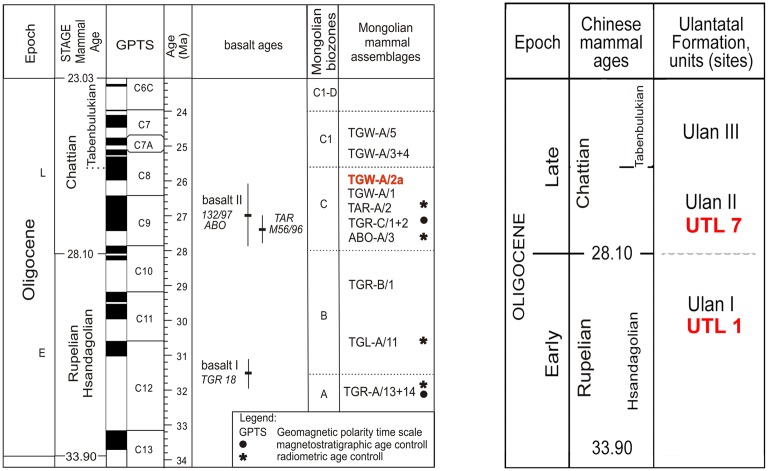
Stratigraphic chart, modified after Daxner-Höck et al. [44, 51, 52]. It includes the geologic time scale [53], basalt ages and Mongolian biozones A to C1-D [44], Mongolian mammal assemblages [53] and magnetostratigraphical data [54]. Stratigraphical scheme for Chinese localities of Ulantatal, data source: Vianey-Liaud et al. [55] and Schmidt-Kittler [47].

In Mongolia, the red beds of the Hsanda Gol Formation (Fm.) can be traced through the Valley of Lakes. Their rich fossil content and several embedded basalt layers—specifically in the Taatsiin Gol and Tatal Gol area—provide a geochronological framework based on the Oligocene Mongolian biozones A, B, C, C1 and C1-D and on the radiometric ages (^40^Ar/^39^Ar) of basalt I and basalt II [43–44]. In the Taatsiin Gol and Tatal Gol area, fossils of biozones A and B discovered below and above basalt I (31.5 ± 0.8 Ma), respectively, suggest an early Oligocene age. Fossils of biozone C are of early late Oligocene age as evidenced by contacts with basalt II. North of the Taatsiin plateau, basalt II of the Unzing Churum section (TAR M56/96; 27.4 ± 0.4 Ma) is situated immediately below the fossil bed TAR-A/2 of biozone C, and basalt II of the Abzag Ovo section (ABO 132/97; 27.0 ± 0.9 Ma) is situated on top of the fossil bed ABO-A/3 of biozone C (Fig 1).

The proposed chronology was recently confirmed by magnetostratigraphical data. Fossil bed TGR-A/13+14 below basalt I of the TGR-A section could be correlated with Chron C12r, and the fossil beds TGR-C/1+2 of the TGR-C section with Chron C9n, respectively [44].

The Mongolian fossil site Toglorhoi (TGW) displays red silty clay of the Hsanda Gol Fm. with several fossil layers of biozones C and C1 along the section TGW-A. The lowermost layer TGW-A/1 of the section is followed by TGW-A/2a+2b, TGW-A/3+4, and on top fossil bed TGW-A/5 was found (Fig 1). Key fossils of biozone C, discovered in layers TGW-A/1 and TGW-A/2a+b, and key fossils of biozone C1 from assemblages TGW-A/3+4 and TGW-A/5 suggest a late Oligocene age.

The fauna of TGW-A/2a comprises fossils of the late Oligocene, in particular characteristic of biozone C: the Lagomorpha *Bohlinotona* cf. *pusilla*, the Eulipotyphla *Amphechinus taatsiingolensis* and the Rodentia *Tataromys sigmodon*, *Tatataromys minor longidens*, *Bohlinosminthus parvulus*, *Eucricetodon bagus* and *Aralocricetodon schokensis*. These fossils are also found in assemblages TAR-A/2, ABO-A/3, TGR-C/1+2 and TGW-A/1. The assemblages from the upper part of the section TGW-A/3+4 and TGW-A/5 differ by characteristic taxa of biozone C1: the Eulipotyphla *Palaeoscaptor gigas* and the Rodentia *Yindirtemys deflexus*. So far, these fossils have been found only in late late Oligocene strata of the region.

Precise stratigraphical correlation of the Mongolian fossil bed TGW-A/2a with the Chinese Ulantatal samples (UTL1 and UTL7) is not possible. According to Vianey-Liaud et al. [55], locality UTL1 and UTL 7 are grouped into biozones Ulan I of the lower Oligocene and Ulan II of the early upper Oligocene, respectively. Daxner-Höck et al. [10] further correlated Ulan I and II from Ulantatal to biozone B and C of Valley of Lakes area, respectively (Fig 1).

## Material and methods

### Institutional abbreviations

NHMW: Naturhistorisches Museum Wien, Vienna, Austria; IVPP: Institute of Vertebrate Paleontology and Paleoanthropology, Beijing, China.

### Material and methods

The studied material includes 17 upper and lower molars, from the late Oligocene locality Toglorhoi (sample TGW-A/2a) of the Taatsiin Gol area (Mongolia), and two lower molars from the Oligocene of Ulantatal (samples UTL1 and UTL 7) in Inner Mongolia (China) described as *Plesiodipus* sp. by Gomes Rodrigues et al. [56]. The anatomical abbreviations for the first, second and third upper molars are M1, M2, and M3 and, similarly, for lower molars, m1, m2, and m3. The studied fossils are stored in the collections of the Geological-Paleontological Department at the Museum of Natural History of Vienna (Austria) and at the Institute of Vertebrate Paleontology and Paleoanthropology, Chinese Academy of Sciences, Beijing (China). They are catalogued under the numbers: NHMW 2015/0312/0001 to 2015/0312/00013 at the MHMW and IVPP V17652.1 and IVPP V17653.1 at the IVPP.

No permits were required for the described study. This material was compared with the descriptions and measurements of the type material of *Argyromys aralensis* from Akespe, North Aral Region, Aral Formation (Kazakhstan). In addition, we revised the description of the *Aralocricetodon* aff. *schokensis* from the Valley of Lakes (Mongolia) and *Aralocricetodon schokensis* [31] from Aral Formation (Kazakhstan) based on the material and casts, respectively, stored at the NHMW. We compared our material with *Plesiodipus leei* [57] from Lanzhou, Gansu, (China) stored at the NHMW and *Plesiodipus wangae* [56] from Ulantatal area stored at the IVPP.

Ulantatal faunas from Inner Mongolia are particularly interesting for comparison purposes. This area provides an excellent example of the faunal assemblages of Central Asia and it is particularly informative for mid-Cenozoic muroids [5, 55–67]. The Ulantatal Formation has provided seven fossil localities from lithostratigraphical units I, II and III [55]. Considering the similarities between the faunal assemblages of Valley of Lakes and Ulantatal, the lithostratigraphical Units I-III were correlated with the biozones B to C1 for the Oligocene of Mongolia [10]. All these new studies of Ulantatal stratigraphy have been recently discussed by Zhang et al. [67], who published the results of the new studies on stratigraphy of the Ulantatal area. During our work, we found some specimens from the localities UTL1 and UTL7 that have strong similarities with our material from Mongolia.

The observations and measurements were carried out using a binocular microscope Zeiss Discovery V20. Maximum length and width measurements for each specimen, given in mm, were taken using Carl Zeiss software Axiocam MRc5 by means a digital camera attached to a microscope. All the measurements are given in Table 1.

**Table 1:** Length and width of the upper and lower molars of *Argyromys* species.

			Length				Width			
Tooth	Locality	Species	Min	Mean	Max	N	Min	Mean	Max	N
**M1**	Akespe	*A*. *aralensis*		2.30		1		2.00		1
	TGW-A/2a	*A*. *cicigei* sp. nov.	2.24	2.29	2.37	3	1.62	1.69	1.76	3
**M2**	Akespe	*A*. *aralensis*		1.95		1		2.00		1
	TGW-A/2a	*A*. *cicigei* sp. nov.		1.80		1		1.66		1
**M3**	TGW-A/2a	*A*. *cicigei* sp. nov.	1.37	1.41	1.46	2	1.39	1.39	1.40	2
**m1**	Akespe	*A*. *aralensis*	2.05	2.30	2.45	3	1.60	1.67	1.75	3
	TGW-A/2a	*A*. *cicigei* sp. nov.	1.94	2.07	2.19	2	1.39	1.48	1.53	3
	UTL1	*A*. *cicigei* sp. nov.		2.10		1		1.30		1
	UTL7	*A*. *cicigei* sp. nov.		1.94		1		1.18		1
**m2**	Akespe	*A*. *aralensis*	2.05	2.15	2.30	3	1.70	1.77	1.85	3
	TGW-A/2a	*A*. *cicigei* sp. nov.	1.93	1.98	2.03	2	1.53	1.56	1.59	2
**m3**	Akespe	*A*. *aralensis*		2.00		1		1.65		1
	TGW-A/2a	*A*. *cicigei* sp. nov.	1.84	1.88	1.91	2	1.47	1.48	1.49	2

*Argyromys aralensis* from Akespe in Kazakhstan, data source: Lopatin [32]; *Argyromys cicigei* sp. nov. from TGW-A/2a, Valley of Lakes (Mongolia) and UTL1 and UTL7, Ulantatal (China). Measurements are in mm. Abbreviations: Min, minimum value; Max, maximum value; N, number of specimens.

**Fig 2:**
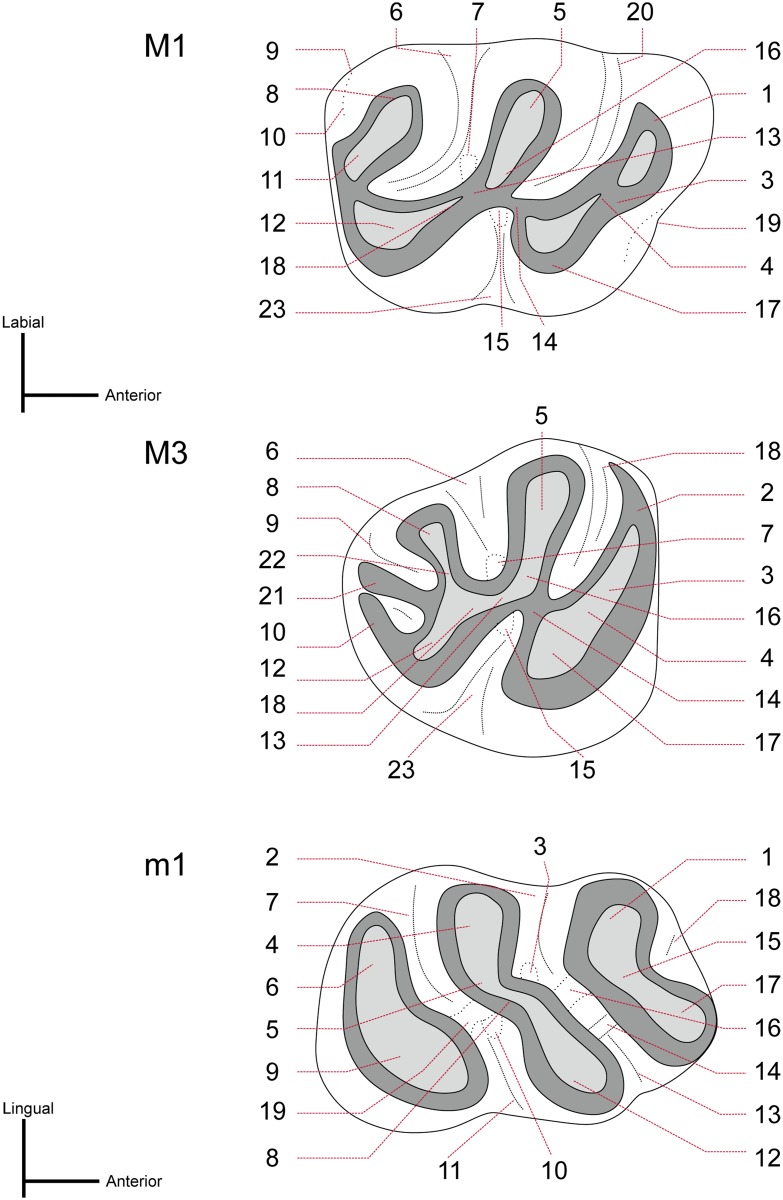
Terminology of the parts of the cheek teeth of *Argyromys*. Only the M1, M3, and m1 morphologies have been drawn; however, the nomenclature can be applied to the rest of the upper and lower molars. Upper molars (M1-M3): 1, anterocone; 2, labial anteroloph 3, anterolophule; 4, anterior arm of the protocone; 5, paracone; 6, mesosinus; 7, mesoloph; 8, metacone; 9, posterosinus; 10, posteroloph; 11, metalophule II (distal); 12, hypocone; 13, entoloph; 14, posterior arm of the protocone; 15, entomesoloph; 16. protolophule II (distal); 17, protocone; 18, anterior arm of the hypocone; 19, protosinus; 20, anterosinus; 21, second posteroloph; 22, metaloph; 23, sinus. Lower molars (m1-m3): 1, metaconid; 2, mesosinusid; 3, mesolophid; 4, entoconid; 5, hypolophulid; 6, posterolophid; 7, posterosinusid; 8, ectolophid; 9, hypoconid; 10, ectomesolophid; 11, sinusid; 12, protoconid; 13, protosinusid; 14, anterolophulid; 15, metalophulid I (proximal); 16, metalophulid II (distal); 17, anteroconid; 18, anterosinusid; 19, anterior arm of the hypoconid.

The measurements of the type material of the species *Argyromys aralensis* from Kazakhstan published by Lopatin [32] are included in Table 1. The terminology used to describe the teeth is taken from Freudenthal et al. [68] and Maridet et al. [15] and summarized in Fig 2.

The photographs were taken with a Philips XL 30 scanning electron microscope at the Core Facility of Cell Imaging and Ultrastructure Research (CIUS) EM LAB Faculty of Life Sciences, University of Vienna (Austria), and Hitachi SEM-3700N in the Key Laboratory of Vertebrate Evolution and Human Origin of Chinese Academy of Sciences, Chinese Academy of Sciences.

The electronic edition of this article conforms to the requirements of the amended International Code of Zoological Nomenclature, and hence the new names contained herein are available under that Code from the electronic edition of this article. This published work and the nomenclatural acts it contains have been registered in ZooBank, the online registration system for the ICZN. The ZooBank LSIDs (Life Science Identifiers) can be resolved and the associated information viewed through any standard web browser by appending the LSID to the prefix “http://zoobank.org/”. The LSID for this publication is: urn:lsid:zoobank.org:act:A7689BC0-A5C0-41FC-AADD-0160D5FDCB54. The electronic edition of this work was published in a journal with an ISSN, and has been archived and is available from the following digital repositories: PubMed Central, LOCKSS.

## Systematic palaeontology

Order Rodentia Bowdich, 1821

Family Cricetidae Fischer, 1817

Genus *Argyromys* Schaub, 1958

**Type species.** *Argyromys aralensis* (Argyropulo, 1939)

*Argyromys cicigei* sp. nov. LSIDurn:lsid:zoobank.org:act:A7689BC0-A5C0-41FC-AADD-0160D5FDCB54

(Figs 3—5; Table 1)

**Fig 3:**
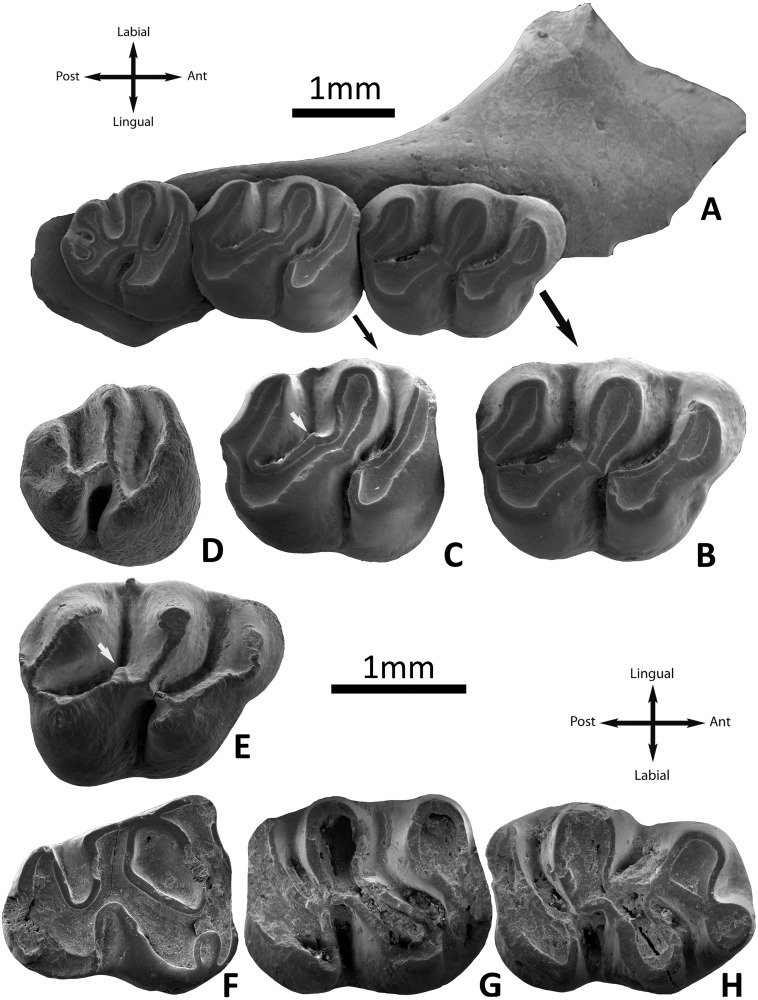
*Argyromys cicigei* sp. nov. from the Valley of Lakes. Toglorhoi locality, section TGW-A, fossil layer TGW-A/2a. A, holotype (NHMW2015/0312/0001 right maxilla); B–C, close-up of the M1 and M2; D, right M3 (NHMW2015/0312/0006); E, inverted left M1 NHMW2015/0312/0003) white arrow indicates the anterior arm of the hypocone; F, inverted left m3 (NHMW2015/0312/0012); G–H, inverted left mandibular, fragment with m1-m2 (NHMW2015/0312/0007). E–H reversed images.

**Fig 4:**
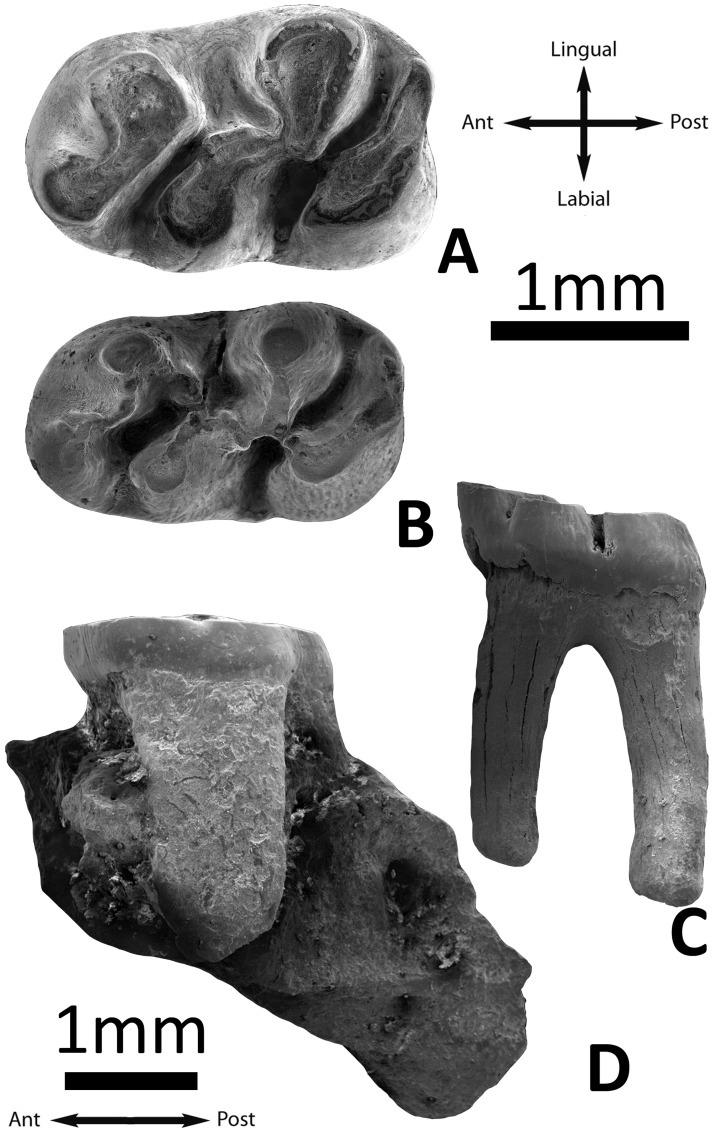
*Argyromys cicigei* sp. nov. from Ulantatal and the Valley of Lakes. A, UTL1 left m1 (IVPP V17652.1); B, UTL7 left m1 (IVPP V17653.1). TGW-A/2a: C, lingual view of the m1 (NHMW2015/0312/0009); D, lingual view of the M1 (NHMW2015/0312/0004).

**Fig 5:**
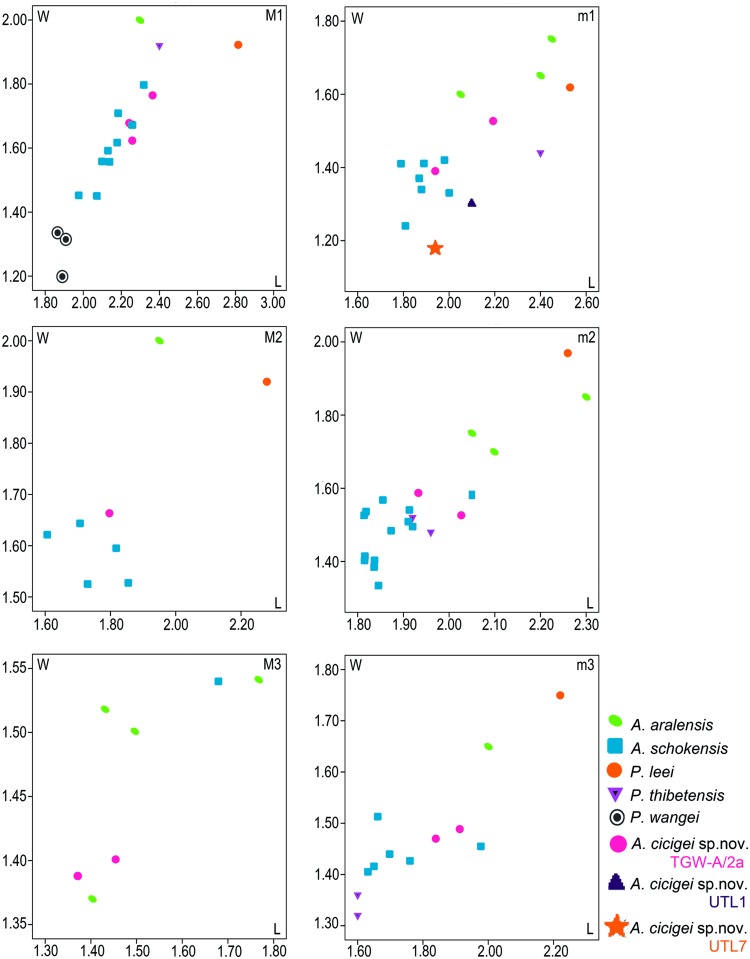
Length/Width scatter-diagram of the cheek teeth of several large-sized species of Cricetidae s.l. Units are given in mm. Source of data: S1 File (*Argyromys cicigei* sp. nov.); Wang [69] (*P*. *thibetensis*); Gomes Rodrigues et al. [56] (*P*. *wangei*); Lopatin [31] (*A*. *aralensis*). Abbreviations: L, length; W, width.

2010 *Aralocricetodon* sp.1 (pro parte); Daxner-Höck, Badamgarav and Erbajeva: 359

2012 *Plesiodipus* sp. Gomes Rodrigues, Marivaux and Vianey-Liaud: 170–171, Fig 6

2014 cf. *Aralocricetodon* sp. Maridet, Daxner-Höck, Badamgarav and Göhlich: 264

**Fig 6:**
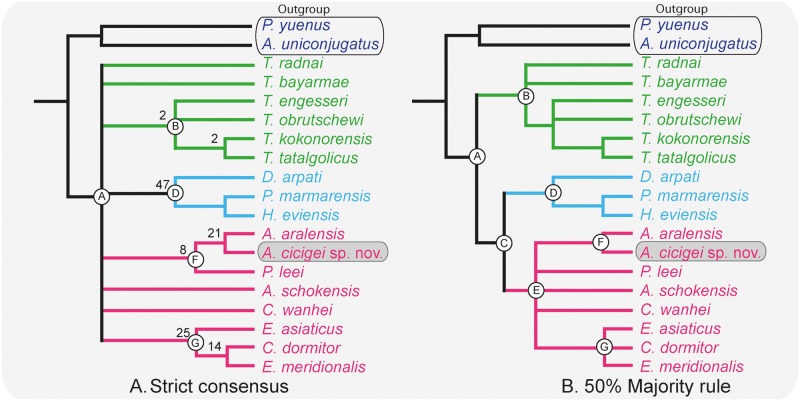
Phylogenetic relationships of *Argyromys* within Tachyoryctoididae, Spalacidae and Cricetidae s.l. (Rodentia). Consensus trees generated from six most parsimonious trees using TNT v. 1.1 [89]. (A) strict consensus, shows a basal polytomy involving the ingroup and four clades can be distinguished, Bootstrapping indices are showed at the appropriate nodes. (B) 50% majority rule consensus, nodes are designated by letters from A to G and displays three main clades. *Argyromys cicigei* sp. nov. from TGW-A/2a and *A*. *aralensis* from Kazakhstan constitute a clade (node F). Node F is grouped with node E that contains the rest of Cricetidae s.l. used in the test (pink). The Spalacidae (blue) and Tachyoryctoididae (green) are grouped in two different clades. The trees have a length of 125 steps, a consistency index (CI) of 0.432 and a retention index (RI) of 0.523.

**Holotype.** NHMW 2015/0312/0001: Fragment of a right maxilla with M1-3.

**Etymology.** Dedicated to Cicige, member of the field team in Mongolia, derived as the genitive of “Cicige”, considered a latinized personal name and ascribed to the fifth declension, according to the rules of Latin grammar (International Commission on Zoological Nomenclature (ICZN), 1999: Article 31.1.1).

**Locality and horizon**. Toglorhoi (TGW), section TGW-A, fossil layer TGW-A/2a. Local biozone C, Chattian, Chinese land mammal age Tabenbulukian, late Oligocene.

**Diagnosis.** Large-sized cricetid rodent with moderate hypsodonty and flat occlusal surface. Sinuses/sinusids not closed by any structure like cingulums or styles. Three-rooted upper molars without mesoloph. M1 with large and undivided anterocone, connected to the protocone and forming a long oblique loph. Protosinus weak or absent. Oblique metalophule II on the upper molars joined to the posterior arm of the hypocone. Posteroloph short or absent on M1 and M2, but well-developed on M3. Posterior arm of the protocone and entoloph on M2 absent; mure (entoloph) deeply interrupted on the M2. Second posteroloph present on M3. Anteroconid on m1 well-developed and displaced labially. Mesoconid absent on m1 and not visible on worn m2 and m3. Metalophulid I on m2 absent. Well-developed lingual anterolophid reaching the protoconid on m2 and m3.

**Differential diagnosis.** *Argyromys cicigei* sp. nov. differs from *A*. *aralensis* by having smaller size; simpler occlusal surface; no spur on the protocone on M1; transversal sinus; absence of mesoloph; no lingual anteroloph and mesocone on M2; no paracone spur on M2; metalophulid II always present on the m1 and no mesolophid on the lower molars.

### Description of the studied material

The upper molars have three roots, two on the labial side and one on the lingual side, which is wider. The lower molars possess two roots. The cusps are stout and rounded and the lophs as well as the enamel are thick. The occlusal surface of the molar is flat after wear. The molars are moderately hypsodont; the crown is high in comparison with the other cricetids from the Oligocene of Mongolia.

#### M1

Material: n = 3. The outline is trapezoidal. The labial valleys and lophs are retroverse. The anterocone is simple and labially placed. The paracone and metacone lack spur or ectolopoh. The anterolophule is slightly oblique and thick; it connects the lingual part of the anterocone with the anterior arm of the protocone. The posterior arm of the protocone is absent in one molar (Fig 3A and 3B) and interrupted in two specimens (Fig 3E). In the latter it is directed towards the lingual extremity of the protolophule II and the long oblique anterior arm of the hypocone, being lower than any of them. The protolophule II is connected to the hypocone. The anterior arm of the hypocone is extremely short and ends freely in the mesosinus (Fig 3E white arrow). The mesoloph and entomesoloph are absent. The posteroloph is weak and distinguishable only in specimens without strong wear. The metalophule II is connected with the short posteroloph near its intersection with the posterior arm of the hypocone. The anterosinus is always open. The protosinus is poorly developed. Only one specimen out of three possesses a cingulum in the mesosinus. The sinus is transversal and open (Fig 3A and 3B).

#### M2

Material: n = 1. The tooth crown is wide with an outline more rectangular than M1. The anterocone is reduced to an enlargement of the enamel on the labial anteroloph. The paracone and metacone are inflated. There is no spur or ectoloph. The anterolophule is slightly oblique and thick; it is merged with the anterior arm of the protocone. The posterior arm of the protocone is absent. The protolophule II is thick; it joins the hypocone. The short anterior arm of the hypocone ends free in the mesosinus (white arrow). The mesoloph and entomesoloph are absent. The metalophule II is present; it is connected to the weak posteroloph. Small platforms are present at the edges of the anterosinus and mesosinus. The anterosinus and mesosinus are open. The protosinus is not developed. The sinus is straight and transversally directed (Fig 3A and 3C). It is connected to the anterosinus by a break between the anterior arm of the protocone and the entoloph.

#### M3

Material: n = 2. The last upper molar shows a rounded outline with a strongly reduced metacone and hypocone. The anterocone is not clearly distinguishable, the labial anteroloph is present and well developed, reaching the labial border. The paracone and metacone are inflated; they do not display any spur or ectoloph. The posterior arm of the protocone is small, short and narrow; it joins the protocone with the protolophule II. The protolophule II is thick; it joins to the anterior arm of the hypocone. The mesoloph and entomesoloph are absent. The metaloph is transversal and joins the anterior arm of the hypocone and the entoloph. The posteroloph is well developed in one specimen; it is short in the other one. The protosinus is not developed. The sinus curves forward. The second posteroloph is present in one tooth; it starts from the posterior arm of the hypocone and it extends through the posterosinus, reaching the posterior edge. One specimen presents a small cingulum in the edge of the anterosinus and the other one possess a small style in the mesosinus (Fig 3D).

#### m1

Material: n = 4. The outline is rectangular. The anteroconid is large, rounded and labially placed. A strong anterolophulid is absent; however, the anteroconid and the protoconid are connected by a weak enamel ridge similar to an anterolophulid in three out of four specimens. The anteroconid and the metaconid are connected by a thick metalophulid I. The metaconid and the anterior arm of the protoconid are connected by the metalophulid II. The mesolophid and the ectomesolophid are absent. The entoconid is connected to the mesio-distal ectolophid and the anterior arm of the hypoconid; sometimes, the latter (Fig 4A) is broken. The hypoconid and entoconid are connected by a small and weak enamel bridge (the anterior arm of the hypoconid) in three out of four teeth. The mesoconid is absent. There is a hypoconulid as a swelling between the posterior arm of the hypoconid and the short posterolophid, the two being merged into a thick posterior curved lophid. The valleys are not closed by cingulids or stylids (Fig 3H).

#### m2

Material: n = 3. The outline is rectangular. The anteroconid is absent. The lingual anterolophid is absent and the labial one is very thick, distinct; it extends towards the protoconid without reaching it. The anterosinusid is narrow. The metalophulid I is wide and connected to the labial anterolophid. A small enamel bridge connects the labial anterolophid with the protoconid. The metalophulid II is absent and the metaconid separated from the ectolophid. The mesoconid, mesolophid and ectomesolophid are absent. The entoconid is connected by the forward directed entolophid to the short ectolophid. The hypoconid displays a posterior arm prolonged into the posterolophid; it is not connected to the entoconid. The hypoconid is linked to the hypolophulid by a short anterior arm. The posterolophid is strong and curved and it does not reach the entoconid. The sinusid and mesosinusid are not closed by cingulids or stylids (Fig 3G).

#### m3

Material: n = 2. Both specimens show a moderate degree of wear obscuring the occlusal morphology. The general morphology is similar to the m2, with the posterior part (hypoconid-entoconid) strongly reduced, forming a subtriangular outline. The anteroconid is absent. The labial anterolophid is not very thick and extends towards the protoconid; it is connected by an anterolophulid, well distinct due to wear. The anterosinusid is small. The metalophulid II, mesolophid and ectomesolophid are absent. The entoconid is connected to the protoconid by an oblique ectolophid directed forwards. There is a weak cingulid joining the metaconid with the entoconid. The posterosinusid is open. An anterior arm of the hypoconid is present in one specimen. The posterolophid is strong and curved and it does not reach the entoconid (Fig 3F).

### Material from Ulantatal section

#### m1

Material: UTL1 (1), UTL7 (1). These molars have two roots. The occlusal surface is flat in the worn specimen from UTL1, and it cannot be assessed in UTL7 because this molar is almost unworn. The outline is sub-rectangular, the anterior width is slightly smaller than the posterior one. The anteroconid is large, rounded and labially placed. The anterolophulid is absent in both specimens; in UTL7, a small piece of enamel is observed between the protoconid and the anteroconid, but it is not big enough to be considered a ridge. The anteroconid and the metaconid are strongly connected by the thick metalophulid I. The metalophulid II in UTL1 appears indistinct, due to wear, but its base is visible at the same place as metalophulid II on the unworn tooth; it is clearly present in the specimen from UTL7, in which it is connected to the posterior branch of the protoconid. The mesolophid and ectomesolophid are absent. They have a longitudinal ectolophid and its central part is enlarged at the place of a mesoconid. The entoconid is connected to the ectolophid. The hypolophulid is transversal. The anterior arm of the hypoconid is connected to the ectolophid in one specimen (UTL7) but it is incomplete in the other one (UTL1). Neither specimen displays a mesoconid but instead a spur or an enlargement of the ectolophid could be considered a sort of mesoconid. The posterolophid is strong and curved; there is a swelling at the place of the hypoconulid. The valleys are deep and open (Fig 4).

## Discussion

The studied samples of fossils from TGW and UTL are morphologically and metrically similar (Table 1, Figs 3–5). The two m1s recovered from Ulantatal section display characters as: the flat occlusal surface on moderately worn specimens; the strong connection between the anteroconid and metaconid, and the small enamel bridges that connect these cusps with the protoconid. These characters are present in TGW-A/2a also. Similarly, they have a posterior weak connection between the entoconid and hypoconid, as the molars from TGW-A/2a. Moreover, the sizes of the m1 of TGW-A/2a and UTL are similar, although the specimen from UTL7 is slightly less wide (Table 1, Fig 5).

Cricetidae from the Taatsiin Gol area were previously studied by Daxner-Höck [39]. Daxner-Höck et al. [10] made a revision of the stratigraphy and biostratigraphy of the Oligocene of Valley of Lakes. They listed several cricetids that were recovered in biozone C: *Aralocricetodon*,? *Eucricetodon* sp.1 and? *Eucricetodon* sp. 2–3. They did not provide information on the distribution of species by localities. The presence of *Aralocricetodon* in biozone C is confirmed, but Daxner-Höck et al. [10] included the material of TGW-A/2a studied here as part of the *Aralocricetodon* collection from biozone C.

Recently Maridet et al. [16] published the Miocene cricetids from the Taatsiin Gol area including a preliminary study of the specimens from the Oligocene. They noted the presence of cf. *Plesiodipus wangae*, *Eucricetodon* sp.2 and *Eucricetodon bagus* in TGW-A/2a. The molars from TGW-A/2a studied here were assigned to *Aralocricetodon* [31] and *Plesiodipus* [57]. We discuss first the generic ascription of the studied fossils.

*Aralocricetodon* was originally described by Bendukidze [31], but new descriptions by Lopatin [32] and Bendukidze et al. [70] improved its morphological information. This genus was also recognized in the Valley of Lakes by Daxner-Höck and Badamgarav [71] and Daxner-Höck et al. [10]. The material studied here differs from *Aralocricetodon* in its simplified occlusal morphology, with a simple anterocone, not split on the M1; lack of mesolophs on the upper molars; a weak posteroloph on the M1 and the absence of a mesolophid and entomesolophid. Nevertheless, *Aralocricetodon* possesses some features such as: high crown and flatted wear surface on all molars; metalophule II on the upper ones; large anteroconid, rounded and labially placed as well as a strong connection between the metaconid and the anteroconid on the m1, which are present in the studied material.

*Plesiodipus* is recovered mainly from localities dated to the middle and early late Miocene of North China (Lierpu in Xining Basin, Quantougou, Tunggur, and Amuwusu) [72–74], although it was recently found in the Oligocene from Ulantatal [56]. Rodrigues et al. [56] noted that the members of this genus possessed a derived pattern of occlusal morphology. *Plesiodipus* has been classified as a member of the subfamily Cricetodontinae [56, 74] or the Gobicricetodontinae [73]. In general, *Plesiodipus* has a more developed protosinus; higher crowns, and wider valleys than the Mongolian material. Its type species, *Plesiodipus leei*, has some similarities with the studied material here such as: low-developed posteroloph on the upper molars; the extremely oblique metalophule and the flat wear [56, 72]. However, it possesses a well-developed protosinus and cingulum on the M1; a paracone spur on the M3; strong anterolophulid and lingual anterolophid on the m1; the lingual anterolophid on the m2 is missing and it is clearly shorter in the Mongolian species (Fig 5). *Plesiodipus wangae* [56] from the Oligocene of Ulantantal, possess similarities with the material from Mongolia such as: an M1 with wear facets nearly flat; posteroloph and the posterosinus missing on the M1 and the missing anterolophulid on the m1. However, it has a well-developed protosinus on the M1; the anterocone presents a small lingual anteroloph; the anterolophule is linked to the labial part of the anterocone on the M1; the sinus is wide; a spine on the hypocone is present in its anterior part and the M1 displays small cingulums on the anterosinus and the mesosinus. Besides, *P*. *wangae* has a well-developed labial anteroloph that is joined to the protoconid on the m1 and the hypolophulid is transversal.

*Argyromys* from Akespe, Kazakhstan [32] shares a number of morphological traits with the Mongolian material. The upper molars have high crowns; flat wear; the protolophule and metaloph strongly developed on the upper molars; a poorly-developed protosinus on the M1; the anterocone on the M1 labially placed; strong connection between the anteroconid and the metaconid on the lower molars; an isolated anteroconid-metaconid complex on the lower molars. Considering of the above mentioned differences with *Aralocricetodon* and *Plesiodipus* and the similarities with *Argyromys*, we have ascribed the material from TGW-A/2a, UTL1, and UTL7 to the genus *Argyromys*.

So far, *Argyromys* is monospecific. It was first described by Argyropoulo [50], and the material of the type species *Argyromys aralensis*, (previously named as *Schaubeumys aralensis*) was re-studied by Lopatin [32]. He provided detailed descriptions, new measurements and drawings. We find several differences between the Kazakhstani assemblage and the fossils studied here. The specimens of *Argyromys* from Mongolia and China are smaller than the Kazakhstani fossils (Fig 5). The upper molars show simpler occlusal surfaces in TGW-A/2a than in the type species. The protocone on the M1 of *Argyromys aralensis* is strongly compressed and the posterior part shows a spur (see Fig 38a in [32]) which is not found in our material. The sinus of the M1 in *A*. *aralensis* is posterolabially directed [32] whereas it is transversal in TGW-A/2a. Our material does not display a clear mesocone or mesoloph which are described in *A*. *aralensis*. The second molar also presents several differences, such as: the M2 from TGW-A/2a does not display lingual anteroloph, neither a mesocone nor a mesoloph as is shown by the figure 38 in Lopatin [32]. Besides, the paracone of the M2 from Kazakhstan possess a small posterior spur [32], which is absent in the Mongolian material. Additionally, the posteroloph of *A*. *aralensis* is more developed than in the specimens from TGW-A/2a. The lower first molars of *A*. *aralensis* present the anteroconid and the metaconid isolated by a deep valley [32], whereas in the m1 from TGW-A/2a and UTL the metalophulid II is always present, joining the metaconid with the ectolophid. Also, the anteroconid and the protoconid are connected by a weak enamel ridge similar to an anterolophulid on the Mongolian and Chinese m1s. The fossils from Kazakhstan possess a so-called pseudomesolophid, composed by a long posterior arm of the protoconid that ends free in the mesosinus [32], whereas in TGW-A/2a and UTL, the posterior arm of the protoconid is connected to the ectolophid. Similarly, the studied material does not display the spur on the entoconid described by Lopatin [32]. The m2 of *A*. *aralensis* possess two anterolophids, whereas the lingual one is always absent in TGW-A/2a. Furthermore, the short mesolophid found in the figures by Lopatin [32] is absent in TGW-A/2a. This lingual anterolophid was also described for the m3 on *A*. *aralensis* and is absent in the TGW-A/2a molars, as well as a mesolophid, which is figured in Lopatin [32]. As a result, the studied material does not display two different mesosinusids, anterior and posterior, as described for *A*. *aralensis*.

After this detailed study and the comparison with all taxa abovementioned, the evidence presented here suggests that the combination of characters in the specimens of Mongolia and China is unique, allowing us to propose the new species *Argyromys cicigei* sp. nov. for the material from TGW-A/2a, UTL1 and UTL7.

## Cladistic analysis

Since its first description in 1958 by Schaub [49], the suprageneric classification of *Argyromys* has been discussed several times [17, 32, 70]. *Argyromys* was included in different groups such as Cricetidae *incertae sedis* [49] or in Cricetidae subfamilies Anomalomyinae [75], Cricetodontinae [76] or Tachyoryctoidinae [21, 70, 71, 77, 78]. In addition, the subfamily Tachyoryctoidinae was included either in the families Rhizomyidae [79], Spalacidae [21], Cricetidae [80] or Muridae [13]. De Bruijn et al. [81] also suggested placing *Argyromys* in a separate family, Tachyoryctoididae. Recently, Wang and Qiu [17] enumerated several characters of *Argyromys*, which are closer to Rhizomyidae representatives and they excluded the genus from the family Tachyoryctoididae.

None of these options were followed by Lopatin [32], who studied the type material from Kazakhstan and concluded that *Argyromys* is an early representative of family Spalacidae. Therefore, the main question is whether *Argyromys* is a member of Cricetidae s.l. or it belongs to Spalacidae (including Rhizomyinae as a subfamily *sensu* Wilson and Reeder [3]). Lopatin [32] commented that the general morphology of *Argyromys* resembles the Spalacids *Heramys* and *Debruijnia*. The characters that, according to Lopatin [32], *Argyromys* shares with *Debruijnia* are: the lophodont pattern; the cuspidate anterocone on the M1; the short mesoloph on the M1; the position of the anteroconid on the m1 and the presence of an anterolingual fold between the anteroconid and protoconid on the m1. These characters are also present in *Aralocricetodon schokensis* from the Aral Formation. *Aralocricetodon* is also present in the Valley of Lakes and Kazakhstan [10, 16, 31, 32, 70]. It has been classified within Cricetodontinae *sensu stricto* [31, 32] and also, as a member of the subfamily Tachyoryctoidinae within the Muridae [70]. The most recent classification was given by Wang and Qiu [17] who included *Aralocricetodon* in the family Cricetidae instead of Tachyoryctoididae, based on the general occlusal structures of the molar and also because *Aralocricetodon* is much smaller in size than the members of Tachyoryctoididae [17].

On the other hand, the main difference that Lopatin [32] found with the cricetids is that *Argyromys* presents a reduced anterocone on the M1. However, Fig 38a of *A*. *aralensis* [32] illustrates the presence of a weak protosinus on the M1 and an individualized anterocone, as large as the paracone or metacone. This is even more obvious in the Mongolian fossils (Fig 3), in which the anterocone is a well-developed cusp.

We acknowledge the overall morphological similarities that *Argyromys* displays with *Heramys* and *Debruijnia*. But the reason for these resemblances is not clear. Many cricetids from the Oligocene present full myomorphy and a strong anterior connection between the protoconid and the metaconid on m1, whereas the spalacids lack a well-developed metalophid [22]. We have no information about the zygomasseteric system of *Argyromys*, hence we cannot assess the degree of myomorphy. Nevertheless, the fragment of the zygomatic plate on the maxilla of TGW-A/2a is short and not inclined (Fig 3A) and we have observed in Mongolian material a weak connection between the protoconid and the metaconid-anteroconid complex (Fig 3), a metalophulid II on the m1s and this structure is well-displayed in *Argyromys* from Kazakhstan [32, 70]. Moreover, the flat occlusal surface in worn specimens; the weakly-developed posteroloph; the oblique protolophule and metaloph on the upper molars, and the lack of a labial anterolophid on the m1 strongly resemble the Miocene genus *Gobicricetodon* and, also, *Aralocricetodon* which are classified as cricetids belonging to the subfamily Cricetodontinae [16, 17]. Hence, we suggest placing *Argyromys* among the Cricetidae s.l. representatives, although more research on this issue needs to be undertaken before the suprageneric assignation of *Argyromys* is more clearly understood. Indeed, the best way to tests this hypothesis is to make a cladistic analysis covering members of Cricetidae s.l. and Spalacidae. In order to decipher the phylogenetic position of *Argyromys cicigei* sp. nov. we performed a phylogenetic analysis to test the hypothesis whether or not *Argyromys* is placed in the same clade as the cricetids s.l. We accordingly selected as the in-group 17 taxa. They represent the families Spalacidae, Tachyoryctoididae and different subfamilies of Cricetidae s.l. from the Oligocene and Miocene from Europe and Central Asia. We included the type species in the genus, *Argyromys aralensis*. The cricetids s.l. are *Cricetops dormitor* [82], *Cricetodon wanhei* [36]; *Eucricetodon asiaticus* [82]; *Plesiodipus leei*, *Aralocricetodon schokensis* and *Eocricetodon meridionalis* [69, 83]. Tachyoryctoididae are *Tachyoryctoides bayarmae* [51]; *T*. *radnai* [52]; *T*. *obrutschewi* [84]; *T*. *tatalgolicus* [85]; *T*. *kokonorensis* [86]; *T*. *engesseri* [17]. We included also the spalacids that Lopatin [32] used to support his hypothesis: *Debruijnia arpati* [87], *Heramys eviensis* [77] and *Pliospalax marmarensis* [88]. Present analysis does not intend to be a complete phylogenetic study of the whole families Spalacidae, Tachyoryctoididae or Cricetidae s.l. because we just focused on several taxa that have been used to support the hypothesis tested here.

Our analysis is based on a morphological data matrix including 56 characters, derived from Maridet and Ni [39]. Not all characters were applicable in the present study, so 48 out of the initial characters were selected. In addition, we introduced eight new characters for the morphology of the taxa mentioned above (see S2 and S3 Files). Data were collected from fossils and photographs of the type specimens as well as from literature. The characters are mainly based on dental morphology and the data matrix has a very low proportion of missing data: only 9.59% of the total cells contain question marks. The intraspecific variation was taken into account and coded as multistate. All characters have equal weight. Characters scored as having multiple states are interpreted as polymorphisms.

We also followed Maridet and Ni [39] and selected the dipodids, *Primisminthus yuenus* [28] and *Allosminthus uniconjugatus* [28], as outgroup in our analysis. TNT (Tree analysis using New Technology) phylogenetic analysis program [89] was used to search for the most parsimonious trees. We performed a run using a traditional search with 1000 replicates with TBR that recovered 35 most parsimonious trees (MPT) of 125 steps (Consistency index (CI): 0.432; Retention index (RI): 0.523) The strict consensus tree (Fig 6A) presents a large basal polytomy involving the species of *Tachyoryctoides*, the clades B and D, that group the members of Spalacidae and Tachyoryctoididae respectively, and the clades G and F, that include the cricetid species. The 50% majority rule resolves the basal polytomy and presents three main clades (Fig 6B).

The distributions of the character states for the internal nodes in the 50% majority rule are detailed in S4 File. The ingroup (node A, Fig 6) is not supported by any synapomorphy. Two big clades are observed on node B and C. The 50% majority rule places all the species of *Tachyoryctoides* in the same clade (node B, Fig 6B) supported by one unambiguous synapomorphy: crest-like anteroconid on the m1. The relationship among the species of *Tachyoryctoides* is not resolved, but they form a monophyletic group. Node C groups all the species of Spalacidae and Cricetidae s.l. used here. It is supported by two unambiguous synapomorphies: the anterior lobe on the M1 is well developed and the protocone posterior arm on the M2 is absent. In both strict consensus and 50% majority rule *Debruijnia arpati*, *Heramys eviensis* and *Pliospalax marmarensis* form a clade supported by four unambiguous synapomorphies (node D, Fig 6B): the moderately hypsodont cheek teeth; the ectolophid (or mure) on the m1 is oblique; the presence of a hypoconid hind arm on the m1 and the oblique shape of the hypoconid in m2. The genus *Argyromys* is grouped with the cricetids s.l., *Aralocricetodon*, *Cricetodon*, *Cricetops*, *Eucricetodon*, *Eocricetodon* and *Plesiodipus* within a clade (node E) supported by three synapomorphies; two are unambiguous: the anterocone developed into a cusp and the protosinus on the M1 is present. Within this clade both species of *Argyromys*, *Argyromys aralensis* and *Argyromys cicigei* sp. nov., form a clade (node F). This node F is supported by six unambiguous synapomorphies: the semi-lophodont occlusal pattern with thick ridges that connect transversally the main cusps; the absent or interrupted posterior arm of the protocone on the M1 and the weak or absent posteroloph on the M1; protosinus on the M1; anterolophulid on the m1 and the labial anterolophid on the m1 are absent. Other cricetid s.l. taxa *Cricetops dormitor*, *Eocricetodon meridionalis* and *Eucricetodon asiaticus* are clustered into a monophyletic group (node G) supported by six synapomorphies, being five ambiguous. The general wear is not flat; the protocone in the M2 is straight (the sinus is not curved forward); the metacone on the M3 is prominent; the lingual anterolophid on the m1 and the hypoconid hind arm on the m2 are present.

## Discussion

The results of the cladistics analysis are clear regarding the suprageneric status of *Argyromys* (Fig 6A and 6B). Both species of this genus are grouped together in both consensus, and 50% majority rule. They are placed in the same clade as *Plesiodipus* in the strict consensus (Fig 6A and 6B). Spalacidae are located in a different clade (node D, Fig 6A and 6B) and Tachyoryctoididae as well (node D, Fig 6A and 6B). In the 50% majority rule, *Argyromys* is placed in the same clade (node E) with those of cricetids s.l. This confirms our hypothesis that *Argyromys* is closer to the cricetids s.l. than to Spalacidae or Tachyoryctoididae. This supports the suggestions made by Kordikova and De Bruijn [80], who included *Argyromys* in Cricetidae. Moreover, the results support De Bruijn et al. [13], who included it in Muridae (= Cricetidae s.l. in this paper). Our analysis precludes the assignation to Spalacidae suggested by Lopatin [32] and also to Tachyoryctoididae suggested by several authors [21, 70, 77, 78]. Species of node D (*Debruijnia arpati*, *Heramys eviensis* and *Pliospalax marmarensis*) differ from the other clades (node E) by the synapomorphies listed in S4 File. It is worth noting that *Argyromys* is grouped with the rest of the Cricetidae s.l., but located in a different clade from *Eocricetodon*, *Eucricetodon* and *Cricetops*, suggesting a different origin.

The similarities between the species of Spalacidae, Tachyoryctoididae and *Argyromys* such as the general flat wear and high crown of cheek teeth, the weak protosinus on the M1, the absence of labial posterolophulid and mesoconid on the m1 and the presence of a labial anterolophid on the m2 are consequently interpreted as the result of convergent evolution. Several authors [13, 21, 90] hypothesized that similar morphologies between these families could be the result of an adaptation to fossorial life-style developed independently and that they had different muroid ancestors. So far, the lack of post-cranial elements in the material of *Argyromys* prevents us to confirm whether it was indeed adapted to fossorial life. However, it is worth noting that the first occurrence of *Argyromys* in the late Oligocene is coeval with the first occurrences of spalacids Tachyoryctoididae [51] in Asia and the diversification pulse of Geomyidae in America [91], two groups of rodents otherwise known for their adaptation to subterranean life. Moreover, as stated by Nevo [92] and Ünay [93], the evolution and diversification of different rodent groups toward subterranean life style could be linked to climatic changes since the Eocene-Oligocene transition, as a way to avoid extreme temperatures and predation. Future investigations of the climatic and environmental changes in Central Asia during the Oligocene compared to the evolutionary pattern of *Argyromys* and Tachyoryctoididae will provide further arguments to test these hypotheses.

## Comment on the age of the assemblages

In the Mongolian Valley of Lakes, several basalt layers outcrop embedded with the Cenozoic sedimentary sequence. The basalts were dated by the ^40^Ar/^39^Ar-method [42–44], Basalt I erupted around 31.5 Ma (range 30.4–32.1, early Oligocene), Basalt II is about 28.0 Ma (range 27.0–28.0Ma, late Oligocene) and Basalt III about 13 Ma (range 12.2–13.2Ma, middle Miocene). The locality studied here, TGW-A/2a, has been placed in the biozone C and it does not have a direct connection to any basalt. However, two other faunas of biozone C were found in section with basalt outcrops. They are: ABO-A/3 (biozone C) immediately below basalt II (^40^Ar/^39^Ar Age 27.0±0.9 Ma) and TAR-A/2 (biozone C) immediately above basalt II (^40^Ar/^39^Ar Age 27.4 ± 0.4 Ma). Therefore, the age of biozone C faunas is ~ 28 Ma and younger. Apart from that, the early/late Oligocene boundary = Rupelian/ Chattian is at 28.1 Ma according to Vandenberghe et al. [52], therefore, the fauna from TGW-A/2a is necessarily of late Oligocene age. According to Vianey-Liaud et al. [55] sample UTL1 would be correlative with the Ulantatal unit Ulan I, and sample UTL7 with unit Ulan II. New studies of Zhang et al. [67] correlate sample UTL1 with unit Ulan I or II, and sample UTL7 with unit Ulan III-IV. However, the Ctenodactylidae found according to Vianey-Liaud et al. [65, 66]—*Tataromys sigmodon*, *Tatataromys minor*, *Yindirtemys ulantatalensis*—from the two samples (UTL1 and UTL7) agree with fossils of biozone C (lower late Oligocene) from Mongolia. Only one *Karakoromys*-tooth hints an early Oligocene resident in sample UTL1.

Bendukidze et al. [70] pointed out that the common presence of *Aralocricetodon schokensis* and *Tachyoryctoides obrutchevi* in Aral Formation (Kazakhstan) and in the assemblages from biozone C and C1 in Mongolia, suggests a correlation of both fauna complexes with the Chinese Tabenbulukian land mammal age (= late Oligocene). The presence of another common taxon like *Argyromys* in both regions, confirms the conclusion of Bendukidze et al. [70] that both regions are closer in age. Bendukidze et al. [70] also state that the differences in faunal composition between Aral Formation and Hsanda Gol from Mongolia may be due to ecological reasons rather than age differences. Our study points out that both regions could have more common elements than previously thought. During the Oligocene, Mongolia, Inner Mongolia and Kazakhstan could be part of a large bioprovince covering most of Central Asia.

## Conclusions

New fossil material of the genus *Argyromys* is found in the Oligocene from Mongolia and North China. Both morphological and metrical traits of the fossils led us to describe a new species, *Argyromys cicigei* sp. nov. The genus was previously only known in Kazakhstan. After the present study, its geographical distribution has greatly increased; *Argyromys cicigei* sp. nov., the representative of the genus in Mongolia and China, is found in the biozone C form the late Oligocene of Mongolia and in the Units Ulantatal I and II from the Oligocene of China. The metrical and morphological analysis presented here adds information about the size and morphology of the genus known by the scarce material from the type locality of *A*. *aralensis* (Akespe). Detailed comparisons with other large-sized cricetids s.l., tachyoryctoidids from the Oligocene of Asia, and spalacids from the Miocene of Europe, allow us to evaluate the suprageneric assignation of *Argyromys*. The controversy about the classification above genus level of *Argyromys* is resolved by our phylogenetic analysis. The strict and 50% majority rule consensus agree in placing the species of *Argyromys* in a monophyletic clade together with cricetid s.l taxa. The spalacids used by Lopatin to conclude that the genus belong to the family Spalacidae, form a different clade. The *Tachyoryctoides* species are grouped together. Our work also suggests that the region of Central Asia comprising Mongolia, China and Kazakhstan could be part of a large bioprovince during the Oligocene.

## Supporting information

S1 File Measurements of the molars of *Argyromys cicigei* sp. nov. (DOCX)

S2 File Character list. (DOCX)

S3 File Character matrix. (DOCX)

S4 File Distributions of the character states for the internal nodes. (DOCX)
