# Supplementary material for: A new species of Argyromys (Rodentia, Mammalia) from the Oligocene of the Valley of Lakes (Mongolia): Its importance for palaeobiogeographical homogeneity across Mongolia, China and Kazakhstan
Source: PLoS One. 2017 Mar 22;12(3):e0172733. doi: 10.1371/journal.pone.0172733 (PMC5362143; doi:10.1371/journal.pone.0172733)
Supplement: S1 File — (DOCX) [file pone.0172733.s001.docx]

Meassurements of *Argyromys cicigei* from Mongolia.

| **Element** | **side** | **length** | **Width** | **Catalogue number** | **Section** | **fossil layer** | *Genus* | *species* |
| --- | --- | --- | --- | --- | --- | --- | --- | --- |
| *max-M1* | d | 2.241 | 1.678 | 2015/0312/0001 | Toglorhoi | TGW-A/2a | *Argyromys* | *cicigei* |
| *max-M2* | d | 1.797 | 1.663 |  | Toglorhoi | TGW-A/2a | *Argyromys* | *cicigei* |
| *max-M3* | d | 1.372 | 1.388 |  | Toglorhoi | TGW-A/2a | *Argyromys* | *cicigei* |
| *M1* | i | 2.259 | 1.622 | 2015/0312/0003 | Toglorhoi | TGW-A/2a | *Argyromys* | *cicigei* |
| *M1* | i | 2.366 | 1.640 | 2015/0312/0004 | Toglorhoi | TGW-A/2a | *Argyromys* | *cicigei* |
| *m2* | d | x | x | 2015/0312/0005 | Toglorhoi | TGW-A/2a | *Argyromys* | *cicigei* |
| *M3* | d | 1.455 | 1.401 | 2015/0312/0006 | Toglorhoi | TGW-A/2a | *Argyromys* | *cicigei* |
| *jaw-m1* | i | 2.193 | 1.526 | 2015/0312/0007 | Toglorhoi | TGW-A/2a | *Argyromys* | *cicigei* |
| *jaw-m2* | i | 2.027 | 1.526 |  | Toglorhoi | TGW-A/2a | *Argyromys* | *cicigei* |
| *m1* | i | x | 1.526 | 2015/0312/0008 | Toglorhoi | TGW-A/2a | *Argyromys* | *cicigei* |
| *m1* | i | 1.94 | 1.39 | 2015/0312/0009 | Toglorhoi | TGW-A/2a | *Argyromys* | *cicigei* |
| *m1* | i | x | x | 2015/0312/0010 | Toglorhoi | TGW-A/2a | *Argyromys* | *cicigei* |
| *m2* | i | 1.934 | 1.587 | 2015/0312/0011 | Toglorhoi | TGW-A/2a | *Argyromys* | *cicigei* |
| *m3* | i | 1.913 | 1.488 | 2015/0312/0012 | Toglorhoi | TGW-A/2a | *Argyromys* | *cicigei* |
| *m3* | i | 1.839 | 1.470 | 2015/0312/0013 | Toglorhoi | TGW-A/2a | *Argyromys* | *cicigei* |
