# Supplementary material for: A new species of Argyromys (Rodentia, Mammalia) from the Oligocene of the Valley of Lakes (Mongolia): Its importance for palaeobiogeographical homogeneity across Mongolia, China and Kazakhstan
Source: PLoS One. 2017 Mar 22;12(3):e0172733. doi: 10.1371/journal.pone.0172733 (PMC5362143; doi:10.1371/journal.pone.0172733)
Supplement: S2 File — (DOCX) [file pone.0172733.s002.docx]

Character list (see below the terminology used to complete the nomenclatural terms on the paper; they are applied to the rest of the taxa of the phyologeny.

1. **Cheek teeth –** brachydont=0; moderately hypsodont=1
2. **Cheek teeth –** bunodont=0; lophodont=1; semi-lophodont (thick connections are fused with the cusps)=2
3. **Cheek teeth, Wear** **–** flat=0; not flat=1
4. **Upper Jaw, Zygomatic plate (Zygoma)** – not inclined (Hystricomorphous)=0; inclined (the anterodorsal tilt of the ventral side of the anterior zygomatic root)=1
5. **Lower Jaw, Diastema** – Shallow and weakly curved=0; deep and strongly curved =1
6. **Lower Jaw, Ventral masseter crest ends** **–** ends under the m1=0; ends under the m2=1
7. **Lower Jaw, axis of teeth row if occlusal view** – strongly inclined compared to the axis of the lower jaw=0; slightly inclined compared to the axis of the lower jaw=1
8. **Upper P4** – absent=0; present=1
9. **Upper M1, Anterior lobe –** not developed=0; developed=1
10. **Upper M1, Anterior protolophule(Protolophule I) –** Absent=0; present (incomplete or complete)=1
11. **Upper M1, Anterocone** **–** developed into cusp=0; crest-like=1
12. **Upper M1, Anterostyle (=Protostyle)** – absent=0; present=1
13. **Upper M1, Posterior arm of the protocone** – absent or interrupted=0; complete=1
14. **Upper M1, Mesolophs** **–** absent=0; present=1
15. **Upper M1, Metacone ridge** – absent=0; present=1
16. **Upper M1, Metaloph** **–** transversal=0; oblique=1
17. **Upper M1, Posterior protolophule (Protolophule II)** **–** Absent=0; incomplete or complete=1
18. **Upper M1, Posterolophs** **–** absent or weak=0; present=1
19. **Upper M1, Protoconule** – absent=0; present=1
20. **Upper M1, Protosinus** **–** absent=0; present=1
21. **Upper M1, roots** **–** three roots=0; four roots=1
22. **Upper M1, Valleys** **–** narrow=0; wide=1
23. **Upper M2, Posterior arm of the protocone** – absent or interrupted=0; complete=1
24. **Upper M2, Metacone ridge** – absent=0; present=1
25. **Upper M2, Posterior protolophule (Protolophule II)** **–** Absent or incomplete=0; complete=1 [ordered]
26. **Upper M2, Protocone oblique (sinus curved forward)** – no=0; yes=1
27. **Upper M2, Protocone posterior arm** – absent=0; present=1
28. **Upper M2, Protoconule** – absent=0; present=1
29. **Upper M3, Metacone**– weak=0; prominent=1
30. **Upper M3, Posteroloph** **–** absent=0; present=1
31. **Upper M3, Second posteroloph** **–** absent=0; present=1
32. **Lower m1, Additional posterior cingulum (differentiated from the posterolophid)** **–** absent=0; present=1
33. **Lower m1, Anterior branch of the hypoconid** **–** absent=0; present=1
34. **Lower m1, Anteroconid** **–** developed into cuspidate=0; crest-like=1
35. **Lower m1, Anterolophulid –** absent=0; present=1
36. **Lower m1, Ectolophid (or mure)** – not oblique=0; oblique=1
37. **Lower m1, Ectolophid** – Absent or incomplete=0; complete=1
38. **Lower m1, Hypoconid hind arm** – always absent=0; present with variability=1
39. **Lower m1, Hypolophulid** – can be interrupted=0; always complete=1
40. **Lower m1, Hypolophulid** – connected on the mesolophid or mesoconid=0; connected on the hypoconid or anteriorly=1
41. **Lower m1, Labial anterolophid** **–** absent=0; present=1
42. **Lower m1, Labial posterolophulid** – absent=0; present=1
43. **Lower m1, Lingual anterolophid** **–** absent=0; present=1
44. **Lower m1, Mesoconid** – absent=0; present=1
45. **Lower m1, Metaconid ridge** – absent=0; present=1
46. **Lower m1, Metalophulid II** **–** absent=0; present=1
47. **Lower m1, Metalophulid I** – always absent=0; present with variability(complete or incomplete)=1
48. **Lower m1, Valleys** – narrow=0; wide=1
49. **Lower m2, Anterior arm of the hypoconid** – absent=0; present=1
50. **Lower m2, Metalophulid I** – absent or interrupted=0; complete=1
51. **Lower m2, Hypoconid hind arm** – absent=0; present=1
52. **Lower m2, Hypoconid oblique** – no=0; yes=1
53. **Lower m2, Hypolophulid** – connected on the mesolophid o mesoconide (or base of the mesolophid)=0; on the hypoconid or anteriorly=1
54. **Lower m2, labial anterolophid** – absent=0; always present=1
55. **Lower m3, Metalophulid I** – can be absent=0; always present=1
56. **Lower m3, Morphology** – similar to m2=0; differentiated (posterior area reduced or simplified)=1

(**A**) M1: 1, anterocone; 2, labial anteroloph; 3, anterolophule; 4, protocone anterior arm); 5, protoconule; 6, paracone; 7, paracone spur; 8, mesosinus; 9, mesostyle; 10, mesoloph; 11, metacone ridge; 12, metacone; 13, posterosinus; 14, posteroloph; 15, metaloph-lophule (metaloph if only one, transverse, is indicated, or metalophule I and metalophule II if two possible positions); 16, mesocone; 17, hypocone; 18, entoloph; 19, protocone distal arm; 20, sinus; 21, lingual cingulum; 22, entomesoloph; 23, mesial and distal protolophules (protolophule I and II); 24, protocone; 25, protocone platform; 26, protostyle spur; 27, protostyle; 28, lingual anteroloph; 29, protosinus; 30, anterosinus. (**B**) m1: 1, metastylid; 2, metaconid; 3, metaconid ridge; 4, protoconid hind arm; 5, mesosinusid; 6, mesostylid; 7, mesolophid; 8, entoconid spur; 9, entoconid; 10, hypolophulid; 11, posterolophid; 12, additional distal cingulid; 13, posterosinusid; 14, hypoconid hind arm; 15, labial posterolophulid; 16, labial posterosinusid; 17, posterior arm of the pronconid and ectolophid; 18, hypoconid; 19, ectomesolophid; 20, sinusid; 21, ectostylid; 22, mesoconid; 23, protoconid; 24, protosinusid; 25, labial anterolophid; 26, anterolophulid; 27, metalophulid; 28, anteroconid; 29, metaconid spur; 30, lingual anterolophid
