# Supplementary material for: A new species of Argyromys (Rodentia, Mammalia) from the Oligocene of the Valley of Lakes (Mongolia): Its importance for palaeobiogeographical homogeneity across Mongolia, China and Kazakhstan
Source: PLoS One. 2017 Mar 22;12(3):e0172733. doi: 10.1371/journal.pone.0172733 (PMC5362143; doi:10.1371/journal.pone.0172733)
Supplement: S3 File — (DOCX) [file pone.0172733.s003.docx]

Character Matrix

| **INGROUP** | **0** | **1** | **2** | **3** | **4** | **5** | **6** | **7** | **8** | **9** | **10** | **11** | **12** | **13** | **14** | **15** | **16** | **17** | **18** | **19** | **20** | **21** | **22** | **23** | **24** | **25** | **26** | **27** | **28** | **29** | **30** | **31** | **32** | **33** | **34** | **35** | **36** | **37** | **38** | **39** | **40** | **41** | **42** | **43** | **44** | **45** | **46** | **47** | **48** | **49** | **50** | **51** | **52** | **53** | **54** | **55** |
| --- | --- | --- | --- | --- | --- | --- | --- | --- | --- | --- | --- | --- | --- | --- | --- | --- | --- | --- | --- | --- | --- | --- | --- | --- | --- | --- | --- | --- | --- | --- | --- | --- | --- | --- | --- | --- | --- | --- | --- | --- | --- | --- | --- | --- | --- | --- | --- | --- | --- | --- | --- | --- | --- | --- | --- | --- |
| *Aralocricetodon schokensis* | 0 | 0 | 0 | ? | ? | ? | ? | 0 | 1 | 0 | 0 | 0 | 1 | 1 | 0 | 1 | 1 | 1 | 0 | 1 | ? | 1 | 1 | 0 | 1 | 1 | 0 | 0 | 0 | 1 | 0 | 0 | 1 | 0 | 0 | 0 | 1 | 0 | 1 | 1 | 0 | 0 | 0 | 1 | 1 | 1 | 1 | 1 | 1 | 1 | 0 | 0 | 1 | 1 | 1 | 1 |
| *Argyromys cicigei* | 0 | 2 | 0 | 0 | ? | ? | ? | 0 | 1 | 0 | 0 | 0 | 0 | 0 | 0 | 1 | 1 | 0 | 0 | 0 | 0 | 1 | 0 | 0 | 1 | 0 | 0 | 0 | 0 | 1 | 1 | 0 | 0 | 0 | 0 | 0 | 1 | 0 | 1 | 1 | 0 | 0 | 0 | 0 | 0 | 1 | 1 | 1 | 0 | 1 | 0 | 0 | 1 | 0 | 1 | 1 |
| *Argyromys aralensis* | 0 | 2 | 0 | 0 | 1 | 0 | 1 | 0 | 1 | 0 | 0 | 0 | 0 | 1 | 0 | 1 | 1 | 0 | 0 | 0 | ? | 1 | 1 | 0 | 1 | 1 | 0 | 0 | ? | ? | ? | 0 | 1 | 0 | 0 | 0 | 1 | 0 | 1 | 1 | 0 | 0 | 0 | 0 | 0 | 0 | 1 | 1 | 1 | 1 | 0 | 0 | 1 | 1 | 1 | 1 |
| *Debruijnia arpati* | 1 | 0 | 0 | ? | ? | ? | ? | 0 | 1 | 0 | 1 | 0 | 1 | 1 | 0 | 0 | 1 | 1 | 0 | 0 | 0 | 0 | 1 | 0 | 0 | 1 | 0 | 0 | 0 | 1 | 1 | 0 | 1 | 0 | 1 | 1 | 1 | 1 | 1 | 1 | 1 | 0 | 1 | 0 | 0 | 1 | 0 | 0 | 1 | 1 | 1 | 1 | 1 | 1 | 0 | 1 |
| *Heramys eviensis* | 1 | 0 | 0 | ? | ? | ? | ? | 0 | 1 | 0 | 1 | 0 | 1 | 1 | 0 | 0 | 1 | 1 | 0 | 0 | ? | 1 | 1 | 0 | 0 | 1 | 0 | 0 | 0 | 1 | 0 | 0 | 1 | 1 | 1 | 1 | 1 | 1 | 1 | 1 | 1 | 0 | 0 | 0 | 0 | 1 | 1 | 1 | 1 | 1 | 0 | 1 | 1 | 1 | 1 | 1 |
| *Pliospalax marmarensis* | 1 | 0 | 0 | ? | ? | ? | ? | 0 | 1 | 0 | 1 | 0 | 1 | 1 | 0 | 1 | 1 | 1 | 0 | 0 | ? | 1 | 1 | 0 | 0 | 1 | 0 | 0 | 0 | 1 | 0 | 0 | 1 | 1 | 0&1 | 1 | 1 | 1 | 1 | 1 | 0 | 0 | 0 | 0 | 0 | 1 | 1 | 1 | 1 | 1 | 0 | 1 | 1 | 1 | 1 | 1 |
| *Plesiodipus leei* | 1 | 2 | 0 | ? | ? | ? | ? | 0 | 1 | 0 | 0 | 0 | 0 | 0 | 0 | 1 | 1 | 0 | 0 | 1 | 1 | 0 | 1 | 0 | 1 | 0 | 0 | 0 | 0 | 0 | 0 | 0 | 1 | 0 | 1 | 1 | 1 | 0 | 1 | 0 | 1 | 1 | 0 | 0&1 | 0 | 0 | 1 | 0 | 1 | 1 | 0 | 0 | 0 | 0 | 1 | 0 |
| *Cricetodon wanhei* | 0 | 0 | 1 | ? | ? | ? | ? | 0 | 1 | 0 | 0 | 0 | 1 | 1 | 0 | 1 | 1 | 1 | 0 | 1 | 1 | 1 | 1 | 1 | 1 | 1 | 0 | 0 | 0 | 1 | 0 | 0 | 1 | 0 | 1 | 0 | 1 | 0 | 0 | 1 | 1 | 1 | 0 | 0&1 | 0 | 1 | 1 | 1 | 1 | 1 | 0 | 0 | 1 | 0 | 1 | 1 |
| *Cricetops dormitor* | 0 | 0 | 1 | 0 | 1 | 1 | 0 | 0 | 1 | 1 | 0 | 0 | 1 | 1 | 1 | 1 | 1 | 1 | 0 | 1 | 0 | 0 | 1 | 1 | 1 | 0 | 0 | 0 | 1 | 0 | 0 | 1 | 1 | 0 | 1 | 0 | 1 | 1 | 1 | 0&1 | 1 | 1 | 1 | 0&1 | 0 | 1 | 1 | 0 | 1 | 1 | 1 | 0 | 0 | 0 | 1 | 0 |
| *Tachyoryctoides bayarmae* | 0 | 0&1 | 0 | 1 | 1 | 1 | 0 | 0 | ? | ? | ? | ? | ? | ? | ? | ? | ? | ? | ? | ? | ? | ? | 1 | 0 | 1 | 1 | 1 | 0 | 0 | 1 | 0 | 0 | 1 | 1 | 0 | 0 | 1 | 0 | 1 | 1 | 1 | 1 | 1 | 0 | 1 | 1 | 1 | 1 | 1 | 1 | 0 | 0 | 0 | 1 | 1 | 1 |
| *Tachyoryctoides radnai* | 0 | 0&1 | 0 | 1 | 1 | 1 | 0 | 0 | 0 | 0 | 1 | 0 | 1 | 1 | 0 | 0 | 1 | 1 | 0 | 0 | 0 | 0 | 1 | 0 | 1 | 1 | 1 | 0 | ? | ? | ? | 0 | 1 | 1 | 0&1 | 0 | 1 | 0 | 1 | 1 | 1 | 0 | 1 | 0 | 0 | 0&1 | 1 | 1 | 1 | 1 | 0 | 0 | 1 | 1 | 1 | 1 |
| *Tachyoryctoides obrutschewi* | 0 | 1 | 0 | 1 | ? | 1 | ? | 0 | 0 | 0 | 1 | 0 | 1 | 0 | 0 | 0 | 1 | 1 | 0 | 0 | 0 | 0 | ? | ? | ? | ? | ? | ? | 0 | 1 | 0 | 0 | 1 | 0 | 1 | 1 | 1 | 0 | 1 | 1 | 1 | 0 | 1 | 0 | 1 | 1 | 1 | 1 | 1 | 1 | 0 | 0 | 1 | 1 | 1 | 1 |
| *Tachyoryctoides tatalgolicus* | 0 | 1 | 0 | 1 | ? | 1 | 0 | 0 | ? | ? | ? | ? | ? | ? | ? | ? | ? | ? | ? | ? | ? | ? | 1 | 0 | 1 | 1 | 1 | 0 | 0 | 1 | 0 | 0 | 1 | 1 | 1 | 1 | 1 | 0 | 1 | 1 | 1 | 1 | 1 | ? | ? | 0 | 1 | 0 | 0 | 1 | 0 | ? | 0 | 1 | 1 | 1 |
| *Tachyoryctoides kokonorensis* | 0&1 | 1 | 0 | 1 | ? | 1 | 0 | 0 | 0 | 0 | 1 | 0 | 1 | 0 | 0 | 0 | 1 | 1 | 0 | 0 | 0 | 0 | 0 | 0 | 1 | 1 | 1 | 0 | 1 | 1 | 0 | 0 | 1 | 1 | 0&1 | 1 | 1 | 0 | 1 | 1 | 1 | 0 | 1 | 0 | 0 | 1 | 1 | 0 | 1 | 1 | 0 | 0 | 1 | 1 | 1 | 1 |
| *Tachyoryctoides engesseri* | 0&1 | 1 | 0 | 1 | ? | 1 | ? | 0 | 0 | 0 | 1 | 0 | 1 | 0 | 0 | 0 | 1 | 1 | 0 | 0 | 0 | 1 | 1 | 0 | 1 | 1 | 1 | 0 | ? | ? | ? | 0 | 0 | 1 | 0 | 1 | 1 | 0 | 1 | 1 | 1 | 0 | 1 | 0 | 0 | 1 | 0 | 1 | 1 | 1 | 0 | 0 | 1 | 1 | 1 | 1 |
| *Eucricetodon asiaticus* | 0 | 1 | 1 | 1 | ? | ? | 0 | 0 | 1 | 0&1 | 0 | 1 | 1 | 1 | 0 | 0 | 0&1 | 1 | ? | 1 | ? | 1 | 1 | 0&1 | 0 | 0 | 0 | ? | 1 | 1 | 0 | 1 | 1 | 0 | 0&1 | 0 | 1 | 1 | 1 | 1 | 1 | 1 | 1 | 1 | 1 | 1 | 1 | 1 | 1 | 1 | 1 | ? | 1 | 1 | 1 | 1 |
| *Eocricetodon meridionalis* | 0 | 0 | 1 | ? | ? | ? | 0 | 0 | 1 | 0 | 0 | 0 | 1 | 1 | 1 | 0 | 0 | 1 | 0 | 1 | ? | 1 | 1 | 0 | 1 | 0 | 1 | 0 | 1 | 0&1 | 0 | 0 | 1 | 1 | 0&1 | 0 | 1 | 0&1 | 1 | 1 | 1 | 0&1 | 1 | 0 | 1 | 1 | 1 | 1 | 1 | 1 | 1 | ? | 0 | 1 | 1 | 0 |
| **OUTGROUP** |  |  |  |  |  |  |  |  |  |  |  |  |  |  |  |  |  |  |  |  |  |  |  |  |  |  |  |  |  |  |  |  |  |  |  |  |  |  |  |  |  |  |  |  |  |  |  |  |  |  |  |  |  |  |  |  |
| DIPODIDAE *Allosminthus uniconjugatus* | 0 | 0 | 0 | ? | ? | ? | ? | 1 | 0 | 1 | 1 | 0 | 0 | 1 | 0 | 0 | 0 | 1 | 1 | 0 | 0 | 0 | 0 | 0 | 0 | 0 | 1 | 0 | 0 | 1 | 0 | 0 | 0 | 0 | 0 | 0 | 0&1 | 0 | 0 | 0 | 0 | 0 | 0 | 2 | 0 | 1 | 0 | 0 | 0&1 | 0 | 0 | 0 | 3 | 1 | 0 | 1 |
| DIPODIDAE *Primisminthus yuenus* | 0 | 0 | 0 | ? | ? | ? | ? | 1 | 0 | 1 | 1 | 0 | 0 | 1 | 0 | 0 | 0 | 1 | 1 | 0 | 0 | 1 | 0 | 0 | 0 | 0 | 1 | 1 | 0 | 1 | 0 | 0 | 1 | 0 | 0 | 0 | 0&1 | 0 | 0 | 1 | 0 | 0 | 1 | 2 | 0 | 1 | 0 | 0 | 1 | 0 | 0 | 0 | 3 | 1 | 0 | 1 |
| DIPODIDAE | 0 | 0 | 0 | ? | ? | ? | ? | 1 | 0 | 1 | 1 | 0 | 0 | 1 | 0 | 0 | 0 | 1 | 1 | 0 | 0 | 0&1 | 0 | 0 | 0 | 0 | 1 | 0&1 | 0 | 1 | 0 | 0 | 0&1 | 0 | 0 | 0 | 0&1 | 0 | 0 | 0&1 | 0 | 0 | 1 | 1 | 0 | 1 | 0 | 0 | 0&1 | 0 | 0 | 0 | 1 | 1 | 0 | 1 |

| **INGROUP** | **30** | **31** | **32** | **33** | **34** | **35** | **36** | **37** | **38** | **39** | **40** | **41** | **42** | **43** | **44** | **45** | **46** | **47** | **48** | **49** | **50** | **51** | **52** | **53** | **54** | **55** |
| --- | --- | --- | --- | --- | --- | --- | --- | --- | --- | --- | --- | --- | --- | --- | --- | --- | --- | --- | --- | --- | --- | --- | --- | --- | --- | --- |
| *Aralocricetodon schokensis* | 0 | 0 | 1 | 0 | 0 | 0 | 1 | 0 | 1 | 1 | 0 | 0 | 0 | 1 | 1 | 1 | 1 | 1 | 1 | 1 | 0 | 0 | 1 | 1 | 1 | 1 |
| *Argyromys cicigei* | 1 | 0 | 0 | 0 | 0 | 0 | 1 | 0 | 1 | 1 | 0 | 0 | 0 | 0 | 0 | 1 | 1 | 1 | 0 | 1 | 0 | 0 | 1 | 0 | 1 | 1 |
| *Argyromys aralensis* | ? | 0 | 1 | 0 | 0 | 0 | 1 | 0 | 1 | 1 | 0 | 0 | 0 | 0 | 0 | 0 | 1 | 1 | 1 | 1 | 0 | 0 | 1 | 1 | 1 | 1 |
| *Debruijnia arpati* | 1 | 0 | 1 | 0 | 1 | 1 | 1 | 1 | 1 | 1 | 1 | 0 | 1 | 0 | 0 | 1 | 0 | 0 | 1 | 1 | 1 | 1 | 1 | 1 | 0 | 1 |
| *Heramys eviensis* | 0 | 0 | 1 | 1 | 1 | 1 | 1 | 1 | 1 | 1 | 1 | 0 | 0 | 0 | 0 | 1 | 1 | 1 | 1 | 1 | 0 | 1 | 1 | 1 | 1 | 1 |
| *Pliospalax marmarensis* | 0 | 0 | 1 | 1 | 0&1 | 1 | 1 | 1 | 1 | 1 | 0 | 0 | 0 | 0 | 0 | 1 | 1 | 1 | 1 | 1 | 0 | 1 | 1 | 1 | 1 | 1 |
| *Plesiodipus leei* | 0 | 0 | 1 | 0 | 1 | 1 | 1 | 0 | 1 | 0 | 1 | 1 | 0 | 0&1 | 0 | 0 | 1 | 0 | 1 | 1 | 0 | 0 | 0 | 0 | 1 | 0 |
| *Cricetodon wanhei* | 0 | 0 | 1 | 0 | 1 | 0 | 1 | 0 | 0 | 1 | 1 | 1 | 0 | 0&1 | 0 | 1 | 1 | 1 | 1 | 1 | 0 | 0 | 1 | 0 | 1 | 1 |
| *Cricetops dormitor* | 0 | 1 | 1 | 0 | 1 | 0 | 1 | 1 | 1 | 0&1 | 1 | 1 | 1 | 0&1 | 0 | 1 | 1 | 0 | 1 | 1 | 1 | 0 | 0 | 0 | 1 | 0 |
| *Tachyoryctoides bayarmae* | 0 | 0 | 1 | 1 | 0 | 0 | 1 | 0 | 1 | 1 | 1 | 1 | 1 | 0 | 1 | 1 | 1 | 1 | 1 | 1 | 0 | 0 | 0 | 1 | 1 | 1 |
| *Tachyoryctoides radnai* | ? | 0 | 1 | 1 | 0&1 | 0 | 1 | 0 | 1 | 1 | 1 | 0 | 1 | 0 | 0 | 0&1 | 1 | 1 | 1 | 1 | 0 | 0 | 1 | 1 | 1 | 1 |
| *Tachyoryctoides obrutschewi* | 0 | 0 | 1 | 0 | 1 | 1 | 1 | 0 | 1 | 1 | 1 | 0 | 1 | 0 | 1 | 1 | 1 | 1 | 1 | 1 | 0 | 0 | 1 | 1 | 1 | 1 |
| *Tachyoryctoides tatalgolicus* | 0 | 0 | 1 | 1 | 1 | 1 | 1 | 0 | 1 | 1 | 1 | 1 | 1 | ? | ? | 0 | 1 | 0 | 0 | 1 | 0 | ? | 0 | 1 | 1 | 1 |
| *Tachyoryctoides kokonorensis* | 0 | 0 | 1 | 1 | 0&1 | 1 | 1 | 0 | 1 | 1 | 1 | 0 | 1 | 0 | 0 | 1 | 1 | 0 | 1 | 1 | 0 | 0 | 1 | 1 | 1 | 1 |
| *Tachyoryctoides engesseri* | ? | 0 | 0 | 1 | 0 | 1 | 1 | 0 | 1 | 1 | 1 | 0 | 1 | 0 | 0 | 1 | 0 | 1 | 1 | 1 | 0 | 0 | 1 | 1 | 1 | 1 |
| *Eucricetodon asiaticus* | 0 | 1 | 1 | 0 | 0&1 | 0 | 1 | 1 | 1 | 1 | 1 | 1 | 1 | 1 | 1 | 1 | 1 | 1 | 1 | 1 | 1 | ? | 1 | 1 | 1 | 1 |
| *Eocricetodon meridionalis* | 0 | 0 | 1 | 1 | 0&1 | 0 | 1 | 0&1 | 1 | 1 | 1 | 0&1 | 1 | 0 | 1 | 1 | 1 | 1 | 1 | 1 | 1 | ? | 0 | 1 | 1 | 0 |
| **OUTGROUP** |  |  |  |  |  |  |  |  |  |  |  |  |  |  |  |  |  |  |  |  |  |  |  |  |  |  |
| DIPODIDAE *Allosminthus uniconjugatus* | 0 | 0 | 0 | 0 | 0 | 0 | 0&1 | 0 | 0 | 0 | 0 | 0 | 0 | 2 | 0 | 1 | 0 | 0 | 0&1 | 0 | 0 | 0 | 3 | 1 | 0 | 1 |
| DIPODIDAE *Primisminthus yuenus* | 0 | 0 | 1 | 0 | 0 | 0 | 0&1 | 0 | 0 | 1 | 0 | 0 | 1 | 2 | 0 | 1 | 0 | 0 | 1 | 0 | 0 | 0 | 3 | 1 | 0 | 1 |
| DIPODIDAE | 0 | 0 | 0&1 | 0 | 0 | 0 | 0&1 | 0 | 0 | 0&1 | 0 | 0 | 1 | 1 | 0 | 1 | 0 | 0 | 0&1 | 0 | 0 | 0 | 1 | 1 | 0 | 1 |
