# Supplementary material for: A new species of Argyromys (Rodentia, Mammalia) from the Oligocene of the Valley of Lakes (Mongolia): Its importance for palaeobiogeographical homogeneity across Mongolia, China and Kazakhstan
Source: PLoS One. 2017 Mar 22;12(3):e0172733. doi: 10.1371/journal.pone.0172733 (PMC5362143; doi:10.1371/journal.pone.0172733)
Supplement: S4 File — (DOCX) [file pone.0172733.s004.docx]

Distributions of the character states for the internal nodes:

NODE A

No synapomorphies

NODE B

33: **0 to 1** Lower m1, Anteroconid – crest-like=1

NODE C

8: **0 to 1** Upper M1, anterior lobe **–** developed=1

26: **1 to 0** Upper M2, Protocone posterior arm – absent=0

NODE D

0: **0 to 1** Cheek teeth, moderately hypsodont=1

35: **0 to 1** Lower m1, Ectolophid (or mure) – oblique=1

37: **0 to 1** Lower m1, Hypoconid hind arm – present with variability=1

51: **0 to 1** Lower m2, Hypoconid oblique – yes=1

NODE E

10: **1 to 0** Upper M1, Anterocone **–** developed into a cusp=0

15: **0 to 1** Upper M1, Metaloph – oblique=1

19: **0 to 1** Upper M1, Protosinus – present=1

NODE F (*Argyromys*)

1: **0 to 2** Cheek teeth – semi-lophodont (thick connections are fused with the cusps)=2

12: **1 to 0** Upper M1, posterior arm of the protocone – absent or interrupted=0

17: **1 to 0** Upper M1, Posterolophs – absent or weak=0

19: **1 to 0** Upper M1, Protosinus – absent=0

34: **1 to 0** Lower m1, Anterolophulid **–** absent=0

40: **1 to 0** Lower m1, Labial anterolophid – absent=0

NODE G

2: **0 to 1** Cheek teeth, Wear **–** not flat=1

25: **1 to 0** Upper M2, oblique (sinus curved forward) – no=0

28: **0 to 1** Upper M2, Metacone– prominent=1

37: **0 to 1** Lower m1, Hypoconid hind arm – present with variability=1

42: **0 to 1** Lower m1, Lingual anterolophid – present=1

50: **0 to 1** Lower m2, Hypoconid hind arm – present=1
